# From Synapses to Circuits, the Role of KIBRA and the WWC Family in Adaptive Brain Function

**DOI:** 10.1111/jnc.70514

**Published:** 2026-07-07

**Authors:** Lenora J. Volk

**Affiliations:** ^1^ Department of Neuroscience UT Southwestern Medical Center Dallas Texas USA; ^2^ Peter O' Donnell Jr. Brain Institute UT Southwestern Medical Center Dallas Texas USA

**Keywords:** AMPA receptor, GABAA receptor, KIBRA, memory, synaptic plasticity, WWC2

## Abstract

*KIBRA* (*WWC1*) has been a subject of scientific interest and investigation for almost two decades following its initial association with nonpathological variation in human memory performance. Work in a variety of animal models confirms that KIBRA supports memory function and demonstrates that regulation of AMPA‐type glutamate receptors is a key mechanism by which KIBRA modulates neuronal function. KIBRA is a scaffolding protein at excitatory synapses, and its interactome is enriched for proteins that regulate AMPA receptor (AMPAR) trafficking and synaptic plasticity as well as neurodevelopmental disorders. Here, I provide a comprehensive discussion of known and potential mechanisms by which KIBRA and its interactome regulate adaptive brain function, encompassing AMPAR trafficking, synaptic plasticity, and experience‐induced modification of circuit dynamics. Disrupted KIBRA function is implicated in a variety of cognitive disorders, and I review mechanisms by which KIBRA may contribute to neuropathology as well as recent work suggesting that KIBRA manipulation may be a target for cognitive enhancement. I expand the discussion to include recent data identifying the KIBRA homolog WWC2 as a regulator of GABA_A_ receptor expression at inhibitory synapses. In contrast to their distinct roles at excitatory and inhibitory synapses, KIBRA and WWC2 promote dendritic arborization in a non‐redundant manner, and I discuss potential shared mechanisms by which WWC proteins regulate neuronal morphology as well as evidence that this function of WWC proteins may be disrupted in neurodevelopmental pathology.

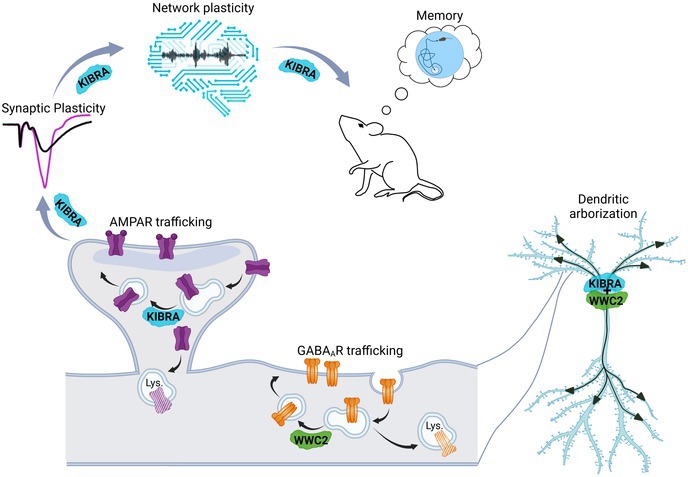

## Graphical abstract

*KIBRA* (*WWC1*) has been the subject of scientific interest since its initial association with nonpathological variation in human memory performance. This review discusses subsequent work in animal models demonstrating that KIBRA supports memory function. The review details progress made towards understanding the molecular, synaptic, and circuit mechanisms by which KIBRA regulates learning and memory, and potential roles for KIBRA in neurodegenerative and neurodevelopmental disorders. KIBRA's role in synaptic plasticity and regulation of AMPA‐type glutamate receptors is highlighted and contrasted with recent work indicating that the KIBRA homolog WWC2 functions as a regulator of GABA_A_ receptor expression at inhibitory synapses. Graphical abstract created in BioRender. Volk (2026) https://BioRender.com/6jbx9t0.

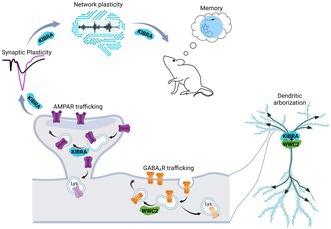

**Abbreviations**
AKAP5: A‐kinase anchoring protein 5
AMOT: angiomotin
AMPARs: α‐amino‐3‐hydroxy‐5‐methyl‐4‐isoxazolepropionic acid‐type glutamate receptors
aPKC: atypical protein kinase C
ASD: autism spectrum disorder
ATM kinase: ataxia‐telangiectasia mutated kinase
AURKA: aurora kinase‐A
CA1, CA3: Cornu Ammonis 1, 3
CAMDI: coiled‐coil protein associated with myosin II and DISC1
CaMKIIα: calcium‐calmodulin‐dependent protein kinase II subunit α
CASK: calcium/calmodulin‐dependent serine protein kinase
CC: coiled coil domain
CDC14 A/B: cell division cycle 14A/14B
CDR: clinical dementia rating
CIT: citron (rho‐Interacting, serine/threonine kinase 21)
(co)‐IP: (co)‐immunoprecipitation
CRB3: crumbs cell polarity complex component 3
DDN: dendrin
DDR1: discoidin domain receptor 1
DN: dominant negative
DNAI1: dynein axonemal intermediate chain 1
DNM1: dynamin 1
DYNLL1,2: dynein light chain 1,2
E/E: excitatory‐excitatory
E/I: excitatory‐inhibitory
EPSC: excitatory postsynaptic current
ERK: extracellular signal‐regulated kinase
FEZ1: fasciculation and elongation protein zeta‐1
FL: full length (protein)
GABA_A_ receptor: ionotropic gamma‐aminobutyric acid receptor
GluA1: AMPA‐type glutamate receptor subunit 1
GluA2: AMPA‐type glutamate receptor subunit 2
GRIP1: glutamate receptor interacting protein 1
GWAS: genome‐wide association study
HAP1: huntington‐associated protein 1
IPSC: inhibitory postsynaptic current
ITC: isothermal titration calorimetry
KI: knockin
KIBRA (WWC1): kidney and brain protein (WW and C2 domain‐containing protein 1)
KIF5B: kinesin family member 5B
KO/cKO: knockout/conditional knockout
LATS1/2: large tumor suppressor1/2
LLPS: liquid–liquid phase separation
LTD: long‐term depression
LTF: long‐term facilitation
LTP/cLTP: long‐term potentiation/chemical long‐term potentiation
MPP2: MAGUK P55 subfamily member 2
MSN: medium spiny neuron
MST1/2: mammalian sterile 20‐like protein kinases 1 and 2
MWM: Morris water maze
MYO6: myosin VI
NCAM1: neuronal cell adhesion molecule 1
NDD: neurodevelopmental disorder
NF2: neurofibromin 2
NMDAR: N‐methyl‐D‐aspartate‐type glutamate receptor
NRXN1: neurexin 1
OE: overexpressed/overexpressing/overexpression
P#: postnatal day #
PACSIN1: protein kinase C and casein kinase substrate in neurons 1
PAR3: par‐3 family cell polarity regulator
PAR6: par‐6 family cell polarity regulator alpha, beta
PATJ: crumbs cell polarity complex component
PFC: prefrontal cortex
pH‐GluA#: pHluorin‐tagged AMPA receptor
PICK1: protein interacting with C kinase 1
PKCι/λ: aPKC isoform: protein kinase C iota/lambda
PKMζ: aPKC isoform: protein kinase M zeta
PP1: protein phosphatase 1
PSD95: postsynaptic density protein 95
PTPN14: protein tyrosine phosphatase non‐receptor type 14
PTPN21: protein tyrosine phosphatase non‐receptor type 21
RISK 1: ribosomal protein S6 kinase alpha‐1
RISK 2: ribosomal protein S6 kinase alpha‐3
SAP97: synapse‐associated protein 97
SCZ: schizophrenia
SEC3: exocyst complex component 1
SEC8: exocyst complex component 4
SEP: super ecliptic pHluorin
SEP‐GluA#: superecliptic pHluorin‐tagged AMPA receptor
SHISA6: shisa protein family member 6
SNP: single nucleotide polymorphism
SNX4: sorting nexin 4
SWR: sharp‐wave/ripple
SYNPO: synaptopodin
TARPγ8: transmembrane AMPA receptor regulatory protein gamma‐8
TS: Tourette Syndrome
WT: wild type
WWC2: WW and C2 domain‐containing protein 2
Y2H: yeast‐two‐hybrid screen
YWHAB, Q, Z: tyrosine 3‐monooxygenase/ tryptophan 5‐monooxygenase activation protein beta, theta, zeta (14–3‐3 beta/alpha, theta, and zeta)
ZDHHC15: zinc finger DHHC‐type palmitoyltransferase

## 1 Introduction

**FIGURE 1:**
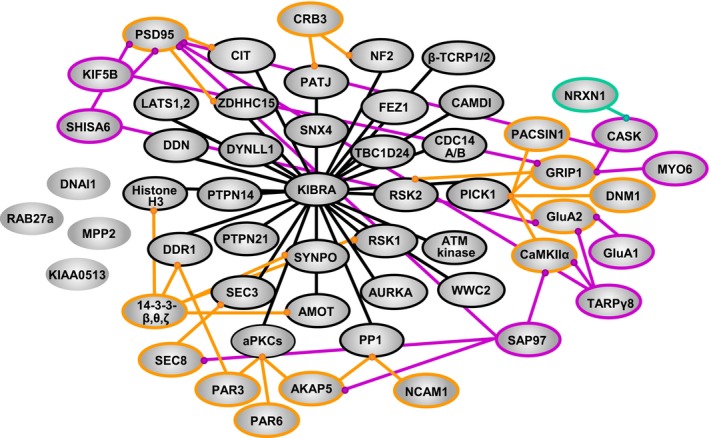
The KIBRA Interactome. Line color indicates binding relationship to KIBRA. Black lines represent direct interaction (see Table 1 for references and mechanisms of interaction). Orange lines represent secondary interactions; proteins in this group interact directly with one or more KIBRA‐binding protein and are found in a complex with KIBRA in cells, but the precise molecular mechanism linking secondary interactors to KIBRA are not established (AKAP5 ‐aPKC (Faux et al. 1999), ‐PP1 (Le et al. 2011); CaMKIIα ‐PICK1 (Lu et al. 2014); CRB3 ‐NF2 (Fan et al. 2025), ‐PATJ (Bhat et al. 1999); DNMN1 ‐PICK1 (Fiuza et al. 2017); GRIP1 ‐PICK1 (Lu and Ziff 2005), RSK2 (Thomas et al. 2005); NCAM1 ‐PP1 (Büttner et al. 2005); PACSIN ‐PICK1 (Anggono et al. 2013); PAR3 ‐aPKCs (Holly et al. 2020; Penkert et al. 2022), DDR1 (Hidalgo‐Carcedo et al. 2011); PAR6 ‐aPKCs (Holly et al. 2020; Penkert et al. 2022); PSD‐95 ‐CIT (Furuyashiki et al. 1999; Zhang et al. 1999), ZDHHC15 (Fukata et al. 2004); SEC8 ‐SEC3 (Matern et al. 2001); 14‐3‐3‐β, θ, ζ ‐AMOT (Centorrino et al. 2022; Adler et al. 2013), DDR1 (Vehlow et al. 2019), Histone H3 (Walter et al. 2008; Winter et al. 2008); RSK1 (Cavet et al. 2003); SYNPO (Hartman et al. 2020; Faul et al. 2008)) or they do not interact directly with KIBRA (GluA2 ‐PICK1 (Xia et al. 1999)). Magenta lines represent tertiary interactions; proteins in this group interact directly with one or more secondary KIBRA interactors and are found in a complex with KIBRA in cells, but the precise molecular mechanism linking these proteins to KIBRA are not established (CASK ‐GRIP (Hong and Hsueh 2006), ‐PSD‐95 (Chetkovich et al. 2002); GluA1 ‐GluA2 (Hansen et al. 2021); KIF5B ‐PSD‐95 (Mok et al. 2002), GRIP1 (Setou et al. 2002); MYO6 ‐GRIP (Lv et al. 2015); SAP97 ‐AKAP5 (Colledge et al. 2000), ‐CaMKIIα (Nikandrova et al. 2010), − PSD‐95 (Cai et al. 2006), ‐SEC8 (Inoue et al. 2006); SHISA6 ‐GluA2 (Klaassen et al. 2016), ‐PSD‐95 (Klaassen et al. 2016); TARPγ8 ‐GluA2 (Tomita et al. 2003), CaMKIIα (Park et al. 2016), PSD‐95 (Zeng et al. 2019)). Cyan lines represent a quaternary interaction, that is, direct binding to a tertiary KIBRA interactor and found in a complex with KIBRA in cells, but the precise molecular mechanism linking this protein to KIBRA is not established (NRXN1 ‐CASK (Hata et al. 1996)). For visual clarity only the closest links to KIBRA are depicted, therefore not all within‐complex interactions are shown. For example, AMOT interacts directly with KIBRA, thus AMOT interactions with NF2, WWC2, PATJ, and LATS1/2 are not depicted in the figure. Likewise, AKAP5 interacts with the KIBRA‐binding proteins PP1 and aPKCs, thus AKAP5 interactions with the secondary and tertiary interactors PSD‐95 and SAP97 are not depicted.

**FIGURE 2:**
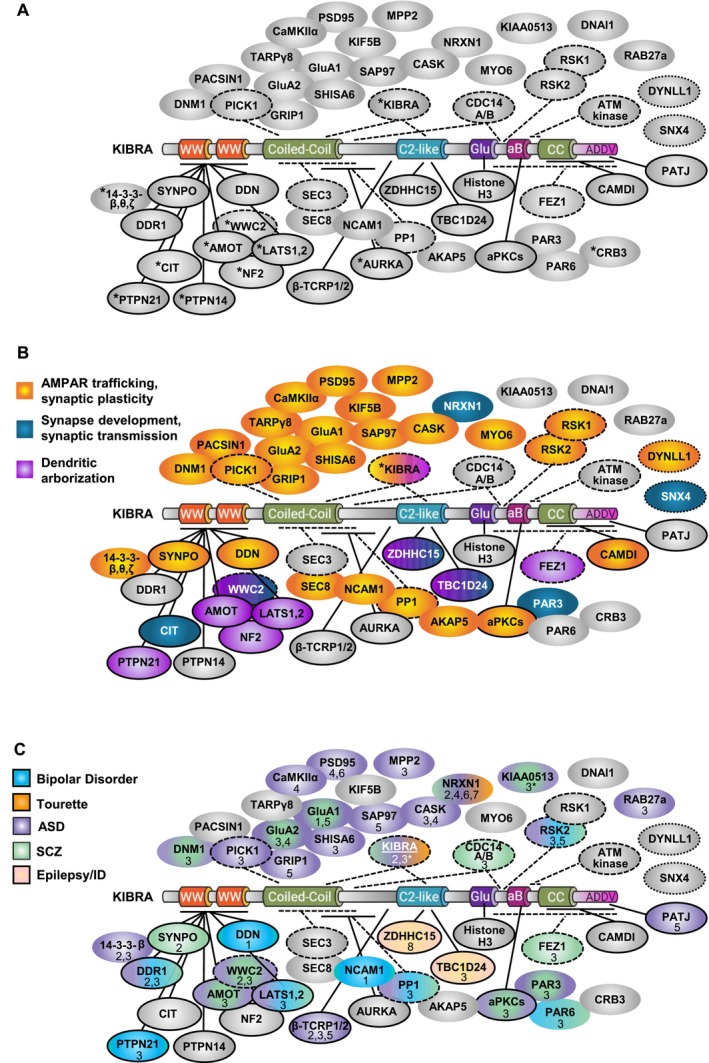
The KIBRA interactome is enriched for proteins that regulate synaptic plasticity, AMPAR trafficking, and brain disorders. (A) KIBRA domain organization and relationship to interactome. Solid lines indicate confirmed binding sites, coarse dashed lines indicate interaction sites inferred from yeast‐two‐hybrid fragment or consensus sequence, fine dashed outline indicates direct interactors with unknown binding site(s), no outline indicates confirmed interaction with KIBRA (e.g., via immunoprecipitation) but mechanism of interaction is unknown or indirect. *Indicates regulator of Hippo signaling pathway. (B) KIBRA interactome components that regulate AMPAR trafficking and/or synaptic plasticity (orange), synapse development and synaptic transmission (teal), and neuronal morphology (purple). (C) The KIBRA interactome is enriched for proteins implicated in neurodevelopmental disorders. Colors indicate the type of neurodevelopmental disorder, numbers indicate the type of study supporting disease association: (1) GWAS, (2) Whole genome or exome sequencing, (3) large genome‐wide expression analysis (3* hub‐gene), (4) high‐confidence ASD_SFARI score 1, (5) strong candidate ASD_SFARI score 2, (6) targeted genetic analysis, (7) large genome‐wide CNV study. See associated references in Table 1.

KIBRA, also known as WWC1, is a scaffolding protein belonging to the WWC family, characterized by common protein–protein and protein–lipid interaction domains (Wennmann et al. 2014; Zhang et al. 2014; Shao and Volk 2025) (Figures 1 and 2A). Work in both humans and animal models has linked KIBRA to memory and cognition. While KIBRA and its binding partners are implicated in several neurological and psychiatric disorders (Willsey et al. 2017; Kos et al. 2016; Kauwe et al. 2024; Tracy et al. 2016; Parikshak et al. 2016) (Figure 2C), polymorphisms in *KIBRA* are also associated with non‐pathological variations in human memory performance (Milnik et al. 2012; Papassotiropoulos et al. 2006; Zhang et al. 2009; Rovira et al. 2016; Schuck et al. 2013). Studies in model systems implicate KIBRA in adaptive brain function at the molecular, synaptic, circuit, and behavioral levels (Makuch et al. 2011; Mendoza et al. 2022; Quigley et al. 2023; Heitz et al. 2016; Vogt‐Eisele et al. 2014; Hu et al. 2017), and both pharmacological and molecular manipulation of KIBRA function show promise as cognitive enhancers (Kauwe et al. 2024; Stepan et al. 2022; Stepan et al. 2024). Thus, current evidence highlights the importance of uncovering the mechanisms by which KIBRA and other WWC proteins affect brain function.

In this review I discuss progress made in understanding the molecular, synaptic, and circuit mechanisms by which KIBRA regulates learning and memory, as well as potential roles for KIBRA and its interactome in neurodegenerative and neurodevelopmental disorders. I also discuss recent data indicating a role for the KIBRA homolog WWC2 in regulating synaptic function and neuronal morphology. Finally, I highlight outstanding questions regarding the mechanisms by which KIBRA and the WWC family of proteins regulate brain function.

## 2 KIBRA Discovery and Basic Protein Features

The first description of KIBRA was provided by Kremerskothen and colleagues while conducting a screen for interactors of the brain‐specific dendritically‐localized protein dendrin (DDN). This study identified a DDN binding partner that was expressed primarily in **KI** dney and **BRA** in, and thus named the protein KIBRA. In the brain, KIBRA expression is enriched in mnemonically important brain structures including the hippocampus and cortex (particularly layer 5) in both humans and rodents (Papassotiropoulos et al. 2006; Quigley et al. 2023; Johannsen et al. 2008). *Kibra* is expressed in most excitatory neurons in the hippocampus and cortex. In contrast, *Kibra* gene expression appears to be restricted to a subset of inhibitory neurons (primarily somatostatin‐positive) with lower expression levels (~3–15 fold lower) in most inhibitory neurons compared to hippocampal CA1/CA3 pyramidal neurons (Quigley et al. 2023). With the exception of astrocytes, *Kibra* expression is not detected in most non‐neuronal cells in the brain (Quigley et al. 2023). However, the cell‐type specificity and relative abundance of KIBRA protein expression have yet to be robustly determined. With respect to KIBRA function in the brain, the vast majority of studies have focused on the role of KIBRA in neuronal physiology, thus little is known about the significance of KIBRA expression in astrocytes.

Structurally, KIBRA contains multiple domains which facilitate interactions with other proteins (Figure 2): (1) N‐terminal WW domains interact with proteins containing PPXY motifs, (2) putative coiled‐coil domains facilitate homo and heterotypic interaction with other coiled‐coil domain‐containing proteins, (3) a C2‐like domain interacts with proteins and lipids (likely in a Ca^2+^‐independent manner), (4) a region that mimics the pseudosubstrate sequence of atypical Protein Kinase C isoforms (aPKCs) serves as a binding site for aPKCs, and (5) a C‐terminal ADDV sequence serves as a ligand for interaction with proteins containing PDZ domains. In addition to the domains mentioned above, which are shared among WWC family proteins, KIBRA also contains a glutamate‐rich stretch near its C‐terminus. Figures 1 and 2A highlight the interactome scaffolded by KIBRA. The finding that AMPA‐type glutamate receptors (AMPARs) and regulators of AMPAR trafficking were components of the KIBRA interactome (Makuch et al. 2011) provided an early mechanistic link between KIBRA and cellular processes that support learning and memory, discussed in more detail in subsequent sections.

## 3 KIBRA in Memory and Cognition

### 3.1 KIBRA in Memory—Human Genetic Studies

Two decades ago, common variants of the *KIBRA* gene were linked to memory performance in healthy human subjects in a Genome Wide Association Study (GWAS) for episodic memory (Papassotiropoulos et al. 2006). This discovery sparked considerable interest in what was at the time a protein of unknown function in the brain. The primary memory‐associated single nucleotide polymorphism (SNP) identified in Papassotiropoulos et al. (rs17070145T/C) resides in an intron, and the effects of this SNP on *KIBRA* gene expression or other aspects of KIBRA regulation are as yet unclear. Two missense SNPs in exon 15 (rs3822660G/T [M734I], rs3822659T/G [S735A]), which encodes KIBRA's C2 domain, were also determined to be in almost complete linkage disequilibrium with rs17070145T/C (Duning et al. 2013). These exonic SNPs are also associated with cognitive performance and may alter C2 domain‐mediated lipid binding and protein interactions (Duning et al. 2013; Posner et al. 2018), though their mechanistic impact on synaptic or circuit function remains to be determined.

The link between *KIBRA* and memory has been observed in many studies (Milnik et al. 2012; Schaper et al. 2008; Preuschhof et al. 2009; Almeida et al. 2008; Vyas et al. 2014; Muse et al. 2014; Kauppi et al. 2011; Yasuda et al. 2010; Vassos et al. 2010; Witte et al. 2016; Bates et al. 2009) and extended to association with cognitive flexibility (Zhang et al. 2009) spatial navigation (Rovira et al. 2016; Schuck et al. 2013), chess mastery and academic achievement (Ahmetov et al. 2023). However, the mnemonic effects of *KIBRA* polymorphisms have not been observed in all studies (Burgess et al. 2011; Need et al. 2008; Wersching et al. 2011; Franks et al. 2014; Korthauer et al. 2018; Homayouni et al. 2024; Boraxbekk et al. 2015) (reviewed in (Schwab et al. 2014; Stepan et al. 2018)). A number of factors may contribute to the variable association of *KIBRA* with memory performance. A meta‐analysis encompassing 8909 subjects from 17 sample populations found that *KIBRA* rs17070145 polymorphism accounts for 0.5% of variance in episodic memory performance (Milnik et al. 2012), suggesting that small sample populations may not have sufficient power to detect the modest effect size for *KIBRA* association with memory. Other factors, including neuropsychiatric disorder status (e.g., schizophrenia, depression, dementia) (Almeida et al. 2008; Vassos et al. 2010; Jurasova et al. 2024; Pantzar et al. 2014) and the presence of other cognition‐modifying gene variants (Jurasova et al. 2024; Pantzar et al. 2014; Porter et al. 2018), may modulate the impact of *KIBRA* SNPs on memory. Finally, in studies that examined the interaction of *KIBRA* genotype, memory performance, and aging, the effect of *KIBRA* rs17070145 on memory increased with advancing age in adulthood (Muse et al. 2014; Kauppi et al. 2011; Stickel et al. 2017; Schuck et al. 2013, 2018) (but see (Bates et al. 2009)). In contrast, *KIBRA* was not associated with memory performance in children and adolescents (Homayouni et al. 2024). Interestingly, synaptic plasticity in juvenile mice was resilient to loss of KIBRA, whereas synaptic plasticity and memory are impaired in adult rodents following loss of KIBRA (Makuch et al. 2011; Mendoza et al. 2022; Heitz et al. 2016; Vogt‐Eisele et al. 2014; Blanque et al. 2015), suggesting that the mnemonic function of KIBRA is influenced by developmental and aging processes.

### 3.2 KIBRA in Memory—Evidence From Animal Models

Work in animal models with specific manipulations of KIBRA expression and function has provided more definitive evidence for KIBRA as a regulator of memory. Constitutive deletion of *Kibra* in mice disrupts associative fear memory (Makuch et al. 2011) and active place avoidance (Vogt‐Eisele et al. 2014). Knockdown of KIBRA in adult rats impairs spatial memory in the Morris water maze (MWM) and increases working and reference memory errors in a radial arm maze (Vogt‐Eisele et al. 2014). Spatial memory in the Barnes maze task is impaired following adult‐induced deletion of *Kibra* from neurons expressing Ca2+/calmodulin‐dependent protein kinase II subunit α (CaMKIIα+ neurons), a population largely composed of excitatory forebrain neurons (Mendoza et al. 2022; Quigley et al. 2023; Erdmann et al. 2007; Allen‐Institute_Cell‐Types 2015; Allen‐Institute_Brain‐Atlas 2004). Spatial memory assessed in the MWM is also impaired in mice engineered to overexpress (OE) KIBRA in CaMKIIα+ neurons (KIBRA OE mice: ~2.5 fold increase in KIBRA protein levels, induced in adult mice) (Heitz et al. 2016). Together these data suggest that KIBRA functions in excitatory neurons to regulate memory storage and use, though the data do not rule out a role for KIBRA in other cell types (inhibitory neurons, astrocytes) (Mendoza et al. 2022; Quigley et al. 2023). Given that both deletion and overexpression of KIBRA impairs memory, these studies also indicate that maintaining KIBRA protein expression levels within a limited physiological range is necessary for optimal mnemonic function.

Additional evidence supporting a role for KIBRA in memory comes from studies using peptides to disrupt KIBRA interaction with binding partners implicated in synaptic plasticity. Viral‐mediated CA1 expression of a peptide designed to block KIBRA‐DDN interaction (“Pb” peptide containing DDN tandem PPXY motifs that bind KIBRA WW domains) results in decreased KIBRA expression in dendritic spines and impaired memory in the Morris water maze (Ji et al. 2019). While it is unclear what proportion of endogenous KIBRA WW domain‐based interactions are disrupted with the DDN Pb peptide, similar plasticity and memory deficits were observed with siRNA‐mediated DDN knockdown, suggesting that disrupted KIBRA‐DDN interaction contributed to the observed synaptic phenotypes. Peptides that block KIBRA interaction with the aPKC isoform zeta (PKMζ) block spatial memory (active place avoidance) when injected into the hippocampus and block associative fear memory when injected into the amygdala (Tsokas et al. 2024). Finally, expression of KIBRA WW domain mutants which enhance KIBRA coupling to AMPAR regulatory complexes improves spatial memory (MWM) and object recognition memory under ‘weak’ training conditions that produce little to no memory in wild type mice (Stepan et al. 2022). Of note, altered memory resulting from manipulation of KIBRA function can persist for weeks (Mendoza et al. 2022; Heitz et al. 2016; Stepan et al. 2022; Tsokas et al. 2024). Though there is some evidence that KIBRA may regulate working memory (Milnik et al. 2012; Vogt‐Eisele et al. 2014) and the speed of learning (Makuch et al. 2011; Mendoza et al. 2022; Stepan et al. 2022), the most robust and consistent effects are observed in tests of long‐term memory.

In summary, there is substantial but not unanimous human genetic data indicating a link between *KIBRA* and adaptive cognition. In contrast, regulation of long‐term memory is consistently observed across a wide variety of methods used to alter KIBRA protein expression and function in rodent models (Makuch et al. 2011; Mendoza et al. 2022; Heitz et al. 2016; Vogt‐Eisele et al. 2014; Stepan et al. 2022; Ji et al. 2019; Tsokas et al. 2024). Adult‐induced disruption of KIBRA function impairs memory (Mendoza et al. 2022; Heitz et al. 2016; Vogt‐Eisele et al. 2014; Ji et al. 2019; Tsokas et al. 2024), indicating that KIBRA plays an active role in mnemonic function independent of KIBRA's role in developmental processes. However, in model organisms the role of KIBRA in memory function has been limited to studies in adult subjects; thus, it is unclear if KIBRA expression during embryonic or early postnatal development is essential for memory function in the immature brain. How aging impacts KIBRA's role in adaptive cognition is an important area for future study.

## 4 KIBRA in Synaptic and Network Plasticity

### 4.1 Synaptic Plasticity

In neurons, KIBRA localization is enriched at excitatory postsynapses (Makuch et al. 2011; Johannsen et al. 2008). Initial clues to the mechanisms by which KIBRA may regulate memory and adaptive cognition came from a study that identified KIBRA as a regulator of hippocampal synaptic plasticity (Makuch et al. 2011). An extensive body of work supports synaptic plasticity, long‐lasting changes in the efficacy of synaptic transmission, as a key cellular mechanism underlying learning and memory (Nabavi et al. 2014; Whitlock et al. 2006; Kandel et al. 2014). Indeed, the KIBRA manipulations which impair or facilitate memory also impair or facilitate synaptic plasticity (Makuch et al. 2011; Mendoza et al. 2022; Heitz et al. 2016; Stepan et al. 2022; Stepan et al. 2024; Ji et al. 2019; Tsokas et al. 2024). Constitutive deletion of KIBRA was shown to impair both synaptic strengthening (Long‐Term Potentiation, LTP) and synaptic weakening (Long‐Term Depression LTD) (Makuch et al. 2011) at excitatory hippocampal Schaffer collateral‐CA1 synapses (SC‐CA1). Subsequent work demonstrated that adult‐induced KIBRA knockdown (Heitz et al. 2016) or knockout (Mendoza et al. 2022) impairs SC‐CA1 LTP, supporting the idea that KIBRA acutely facilitates LTP in mature neurons independent of potential functions in brain development. Further evidence that KIBRA plays an active role in LTP expression comes from work using peptides to acutely disrupt interaction of KIBRA with binding partners DDN (Ji et al. 2019) or PKMζ (Tsokas et al. 2024), both of which impair LTP. In addition, acute pharmacological treatment with a MST1/2 inhibitor increases KIBRA interaction with AMPAR regulatory complexes and potentiates synaptic transmission (Stepan et al. 2024).

KIBRA overexpression also modifies synaptic plasticity (Heitz et al. 2016). Utilizing Tet‐On transgenic mice to generate adult‐induced overexpression of KIBRA in excitatory forebrain (CaMKIIα+) neurons decreases the threshold for LTP induction and accelerates saturation of LTP. A 1 Hz stimulation that typically produces LTD in wild type SC‐CA1 synapses results in LTP in KIBRA OE mice. Consistently, a single 100 Hz tetanus produces larger but saturated LTP in KIBRA OE mice, such that increasing the number of 100 Hz tetanus repetitions further enhances LTP in wild type mice but not KIBRA OE mice, with smaller maximal LTP magnitude in KIBRA OE mice. This disrupted stimulus‐plasticity response is associated with impaired memory in KIBRA OE mice, highlighting the importance of maintaining KIBRA expression levels within physiological boundaries (Heitz et al. 2016).

While the vast majority of studies examining KIBRA's role in synaptic plasticity have focused on hippocampal SC‐CA1 synapses in mammals, KIBRA also regulates synaptic plasticity (associative long‐term facilitation, LTF) in the marine mollusk *Aplysia* (Hu et al. 2017), indicating that KIBRA's role in regulating synaptic plasticity is conserved across species.

As detailed above, the role for KIBRA in long‐term synaptic strengthening (LTP, LTF) is supported by numerous studies using a variety of methods and models to manipulate KIBRA function. However, while loss or overexpression of KIBRA has been shown to impair LTD (Makuch et al. 2011; Heitz et al. 2016), KIBRA's role in LTD has not been as extensively studied. To what extent and under which conditions LTD is regulated by KIBRA is an important topic for further investigation.

Notably, the requirement for KIBRA in synaptic plasticity is differentially regulated during postnatal development. In contrast to the impaired synaptic plasticity observed in adult constitutive KIBRA KO mice (2–3.5 months old) (Makuch et al. 2011), synaptic plasticity was unaffected in juvenile KIBRA KO mice (3–4 weeks old) (Makuch et al. 2011; Blanque et al. 2015) despite similar KIBRA protein expression levels in WT mice across juvenile and adult ages (Mendoza et al. 2022). The resilience of juvenile synaptic plasticity to KIBRA deletion was initially hypothesized to result from compensatory upregulation of the KIBRA homolog WWC2, as WWC2 protein expression was increased in the juvenile but not the adult hippocampus of KIBRA KO mice (Makuch et al. 2011). This hypothesis was directly tested in a subsequent study that employed inducible KIBRA deletion to examine LTP in juvenile and adult mice in the absence of potential developmental confounds (Mendoza et al. 2022). LTP was unaffected when KIBRA deletion was induced in juvenile mice under conditions in which WWC2 is not upregulated (CaMKIIα‐CreER^T2^‐dependent KIRBA deletion induced by tamoxifen injection from P14‐P16, LTP evaluated at P21‐P25). Using the same method, adult‐induced KIBRA deletion impaired hippocampal LTP and memory (Mendoza et al. 2022), indicating that compensatory upregulation of WWC2 is unlikely to account for intact juvenile LTP in the absence of KIBRA. Supporting this conclusion, a recent study demonstrated that WWC2 preferentially localizes to inhibitory rather than excitatory synapses, and that WWC2 does not regulate AMPAR expression (Dunham et al. 2024). Thus, despite similar levels of protein expression in juvenile and adult mice, KIBRA's role in LTP appears to change across postnatal maturation, with a more pronounced role in the adult brain. Interestingly, synaptic plasticity in the juvenile brain is also resilient to deletion of individual AMPAR subunits or PICK1, an AMPAR‐binding protein that interacts with KIBRA (Cao et al. 2018; Jensen et al. 2003; Volk et al. 2010), suggesting that juvenile neurons possess distinct mechanisms to facilitate AMPAR‐dependent plasticity that are not present in adult neurons.

In contrast to KIBRA's role in synaptic plasticity, KIBRA is generally not required for maintenance of basal synaptic transmission. Knockout or overexpression of KIBRA does not affect measures of presynaptic, AMPAR‐mediated postsynaptic, or combined synaptic transmission (Makuch et al. 2011; Mendoza et al. 2022; Heitz et al. 2016)., Likewise, basal synaptic transmission is unaffected by acute disruption of KIBRA‐PKMζ interaction (Tsokas et al. 2024). However, peptide‐mediated disruption of KIBRA‐DDN interactions does decrease basal excitatory synaptic strength at SC‐CA1 synapses (Ji et al. 2019). This apparent discrepancy may be due to the fact that acute expression of the DDN PPXY tandem peptide redistributes KIBRA rather than simply decreasing KIBRA expression, resulting in a dramatic decrease in membrane‐localized KIBRA coincident with sequestration of AMPARs away from the plasma membrane (Ji et al. 2019) (see additional discussion in Mechanisms section).

### 4.2 Network Plasticity

In agreement with its role in synaptic plasticity and memory, KIBRA is essential for experience‐induced changes in hippocampal and cortical network dynamics that support memory consolidation, specifically hippocampal sharp‐wave/ripples (SWRs) and cortical sleep spindles (Quigley et al. 2023). SWRs are brief (50–200 ms), high‐frequency (125–300 Hz) oscillations that occur during offline states such as slow‐wave sleep and quiet wakefulness (Buzsáki 2015; Wilson and McNaughton 1994; Foster and Wilson 2006). SWRs coordinate “replay” or reactivation of sequential neural activity representing awake behavior (Foster and Wilson 2006; Gillespie et al. 2021; Diba and Buzsáki 2007). Substantial evidence supports a causal role for SWRs as a network‐level mechanism for memory consolidation and/or retrieval; disrupting SWRs impairs memory (Girardeau et al. 2009; Ego‐Stengel and Wilson 2010; Jadhav et al. 2012; Gridchyn et al. 2020) whereas prolonging SWRs enhances memory (Fernandez‐Ruiz et al. 2019). Cortical networks also produce brief oscillatory events during slow‐wave sleep termed spindles (0.5–3 s, 10‐15 Hz) which are associated with cortical plasticity and memory consolidation (Novitskaya et al. 2016; Maingret et al. 2016; Rosanova and Ulrich 2005; Werk et al. 2005). Hippocampal SWRs and cortical spindles become more tightly coupled during post‐learning sleep (Novitskaya et al. 2016; Siapas and Wilson 1998; Staresina et al. 2015), and coordinated interaction between the hippocampus and cortex during SWR and spindle events has been proposed as a network mechanism to support information transfer and systems‐level memory consolidation (Siapas and Wilson 1998; Jiang et al. 2019). Consistent with this model, disrupting SWR‐spindle coupling impairs memory consolidation whereas enhancing association of these network events improves memory (Novitskaya et al. 2016; Maingret et al. 2016; Siapas and Wilson 1998; Jiang et al. 2019). Using in vivo electrophysiology in freely‐behaving mice, a recent study showed that KIBRA expression in excitatory forebrain neurons is required for experience‐induced changes in hippocampal SWRs, specifically changes associated with organization and incorporation of new information content in SWRs, including increases in SWR power and duration, increased SWR‐associated low gamma power, and increased participation of CA1 place cells in post‐experience SWRs (Quigley et al. 2023). Additionally, experience‐induced increases in coupling of hippocampal SWRs with prefrontal cortex (PFC) spindles were absent in KIBRA cKO mice. In contrast, basal PFC and hippocampal network dynamics, recorded from naïve mice in their home cage, were largely unaffected, in line with data showing that basal synaptic transmission is intact following loss of KIBRA. Because KIBRA was selectively deleted from excitatory (CaMKIIα+) neurons (Quigley et al. 2023), and considering that KIBRA is enriched at excitatory synapses and does not appear to regulate inhibitory ionotropic GABA_A_ receptors (Makuch et al. 2011; Dunham et al. 2024), these findings elicit a hypothesis wherein KIBRA‐dependent synaptic plasticity at excitatory➔excitatory (E–E) synapses in the hippocampus and/or prefrontal cortex underlie experience‐induced changes in SWR‐ and spindle‐based information processing.

Together, these findings indicate that KIBRA signaling is critical for plasticity at both the synaptic and network levels, whereas it appears to be dispensable for basal synaptic transmission and network function. Future studies using pharmacology or domain mutants to selectively alter KIBRA coupling to specific synaptic signaling mechanisms (e.g., AMPAR trafficking) will be valuable for providing mechanistic links between KIBRA's function at the cellular and network level. While studies of synaptic plasticity in the mammalian brain have focused almost exclusively on the hippocampus, gene expression and network plasticity analyses suggest that KIBRA is likely to play a role in experience‐dependent modification of cortical synapses as well. Finally, KIBRA's role in synaptic plasticity is developmentally regulated, such that the juvenile brain is resilient to loss of KIBRA while KIBRA deletion, overexpression, and disrupting KIBRA coupling to synaptic protein complexes all disrupt synaptic plasticity in the adult brain. Hippocampal network plasticity is also impaired following adult‐induced KIBRA deletion, but it is not known if KIBRA has a developmental role in circuit maturation or function. As discussed below, KIBRA likely plays a role in proper development of neuronal morphology, which might be expected to impact circuit connectivity and function.

## 5 Mechanisms of Synaptic Regulation by KIBRA

The previous sections described the consequences of altered KIBRA expression or *KIBRA* polymorphisms on cognitive function, plasticity, and network adaptation to experience. These studies strongly implicate KIBRA as a key component of plasticity at excitatory synapses. Below, I explore what is known regarding the mechanisms through which KIBRA impacts synaptic plasticity.

### 5.1 KIBRA in AMPAR Trafficking

Modulation of AMPAR trafficking is a conserved mechanism underlying the expression of synaptic plasticity across many cell types and species (Shepherd and Huganir 2007; Diering and Huganir 2018). The KIBRA interactome is highly enriched for proteins that regulate AMPAR trafficking and function (Figure 2B). KIBRA forms a complex with endogenous AMPAR subunits GluA1 and GluA2 in the brain (Makuch et al. 2011; Heitz et al. 2016; Fukuda et al. 2019) and with AMPARs expressed in heterologous cells (Shao and Volk 2025; Heitz et al. 2016). KIBRA can interact with AMPAR subunits through multiple mechanisms. The GluA2‐binding protein PICK1 (Xia et al. 1999) links KIBRA to GluA2‐containing AMPARs (Shao and Volk 2025; Makuch et al. 2011). Whereas KIBRA does not bind directly to GluA2 (Shao and Volk 2025), KIBRA can interact with GluA1 expressed in heterologous cells in the absence of additional AMPAR‐binding proteins (Shao and Volk 2025; Heitz et al. 2016). These data suggest that KIBRA may interact directly with GluA1, though additional studies are necessary to determine how KIBRA binds GluA1.

### 5.2 Basal AMPAR Trafficking

Current evidence indicates that KIBRA promotes basal recycling of GluA1 to the plasma membrane; KIBRA knockdown in cultured neurons decreases GluA1 recycling whereas KIBRA overexpression increases GluA1 recycling back to the plasma membrane following constitutive internalization (Heitz et al. 2016). In addition, adult‐induced KIBRA deletion depletes extrasynaptic AMPARs (measured via total and membrane GluA1 and GluA2) with no effect on synaptic AMPAR content. In contrast to these relatively acute manipulations, constitutive deletion of KIBRA in KIBRA KO mice does not decrease AMPAR expression in total brain homogenate (Makuch et al. 2011) or hippocampal membrane fractions (Stepan et al. 2022), suggesting that homeostatic mechanisms may normalize basal AMPAR expression following persistent loss of KIBRA protein.

### 5.3 Activity‐Induced AMPAR Trafficking and Expression

Despite alterations in basal trafficking and extrasynaptic expression of AMPARs, basal synaptic transmission is largely unaffected by deletion or overexpression of KIBRA (Makuch et al. 2011; Mendoza et al. 2022; Heitz et al. 2016), indicating that neurons are able to maintain functional synaptic AMPAR content (Mendoza et al. 2022) under low activity conditions in the absence of KIBRA. In contrast, KIBRA is critical for activity‐induced AMPAR trafficking and synaptic plasticity (Makuch et al. 2011; Mendoza et al. 2022; Heitz et al. 2016). Using exogenously‐expressed GluA2 tagged with a pH‐sensitive fluorescent reporter to visualize surface receptors, neurons cultured from KIBRA KO mice showed faster recycling of AMPARs back to the plasma membrane following NMDA‐induced internalization (Makuch et al. 2011), prompting the initial hypothesis that KIBRA functions to retain internalized GluA2‐containing AMPARs in a manner functionally similar to the KIBRA binding partner PICK1 (Lin and Huganir 2007). However, subsequent work revealed that pH‐GluA2 may not be a reliable indicator of exclusively surface‐localized AMPARs (Rathje et al. 2013) under these conditions, confounding interpretation of receptor recycling data obtained using this particular assay. Specifically, intracellular pH‐GluA2 contributes to fluorescence signal and bath application of NMDA produces substantial intracellular acidification such that loss of fluorescence signal is not due primarily to receptor internalization. Therefore, while it is plausible that KIBRA can promote retention of internalized AMPARs through its interaction with PICK1 (this mechanism of trafficking regulation has been unambiguously demonstrated for PICK1 through multiple methodologies (Citri et al. 2010; Terashima et al. 2008; Nakamura et al. 2011)), studies using complementary methodology are necessary to determine the contribution of KIBRA to intracellular retention of GluA2‐containing AMPARs. KIBRA was also shown to promote chemical LTP (cLTP)‐induced increases in surface GluA1, as measured by live imaging of exogenously‐expressed SEP‐GluA1 in neurons overexpressing KIBRA (Heitz et al. 2016). This assay is less prone to contamination by the artifacts mentioned above, and is consistent with data demonstrating that pharmacologically enhancing KIBRA association with AMPAR regulatory complexes increases surface expression of endogenous GluA1 and GluA2 as measured by surface biotinylation in brain organoids (Stepan et al. 2024).

In addition to the above studies in cultured neurons, KIBRA has also been shown to play an essential role in LTP‐induced upregulation of endogenous AMPAR expression in the intact hippocampal circuit. LTP increases total GluA1 and GluA2 protein expression in hippocampal CA1 tissue from wild type mice but not from mice with adult‐induced KIBRA deletion (Mendoza et al. 2022). Whether the observed increase in AMPAR expression results from LTP‐induced *de novo* protein synthesis or a decrease in AMPAR degradation (e.g., decreased trafficking to lysosomes) is not yet known.

Finally, disrupted KIBRA function may contribute to aberrant activity‐induced AMPAR trafficking under pathological conditions. In patients with neurodegenerative tauopathies (Alzheimer's and Pick's diseases), decreased KIBRA expression is associated with poorer Clinical Dementia Rating (CDR). Related animal models of tauopathies exhibit impaired activity‐induced AMPAR trafficking (Kauwe et al. 2024; Tracy et al. 2016). Notably, impaired LTP‐induced GluA1 trafficking in these models can be rescued by KIBRA overexpression (full‐length or a C‐terminal fragment of KIBRA) (Kauwe et al. 2024; Tracy et al. 2016), indicative of a potential role for KIBRA in synaptic dysregulation in neurodegenerative disorders.

### 5.4 Mechanisms of KIBRA‐Regulated AMPAR Trafficking

Subunit‐specific AMPAR binding partners play a key role in AMPAR trafficking and synaptic plasticity (Anggono and Huganir 2012). KIBRA can interact with AMPAR subunits GluA1 and GluA2 through distinct mechanisms (Shao and Volk 2025) raising the possibility that KIBRA may be able to affect AMPAR trafficking and function in a subunit‐specific manner. While KIBRA has been shown to regulate both GluA1 and GluA2 (Mendoza et al. 2022; Heitz et al. 2016; Fukuda et al. 2019), whether KIBRA can differentially affect AMPAR trafficking based on AMPAR subunit composition or the specific constituents of the KIBRA protein complex remain important open questions.

**FIGURE 3:**
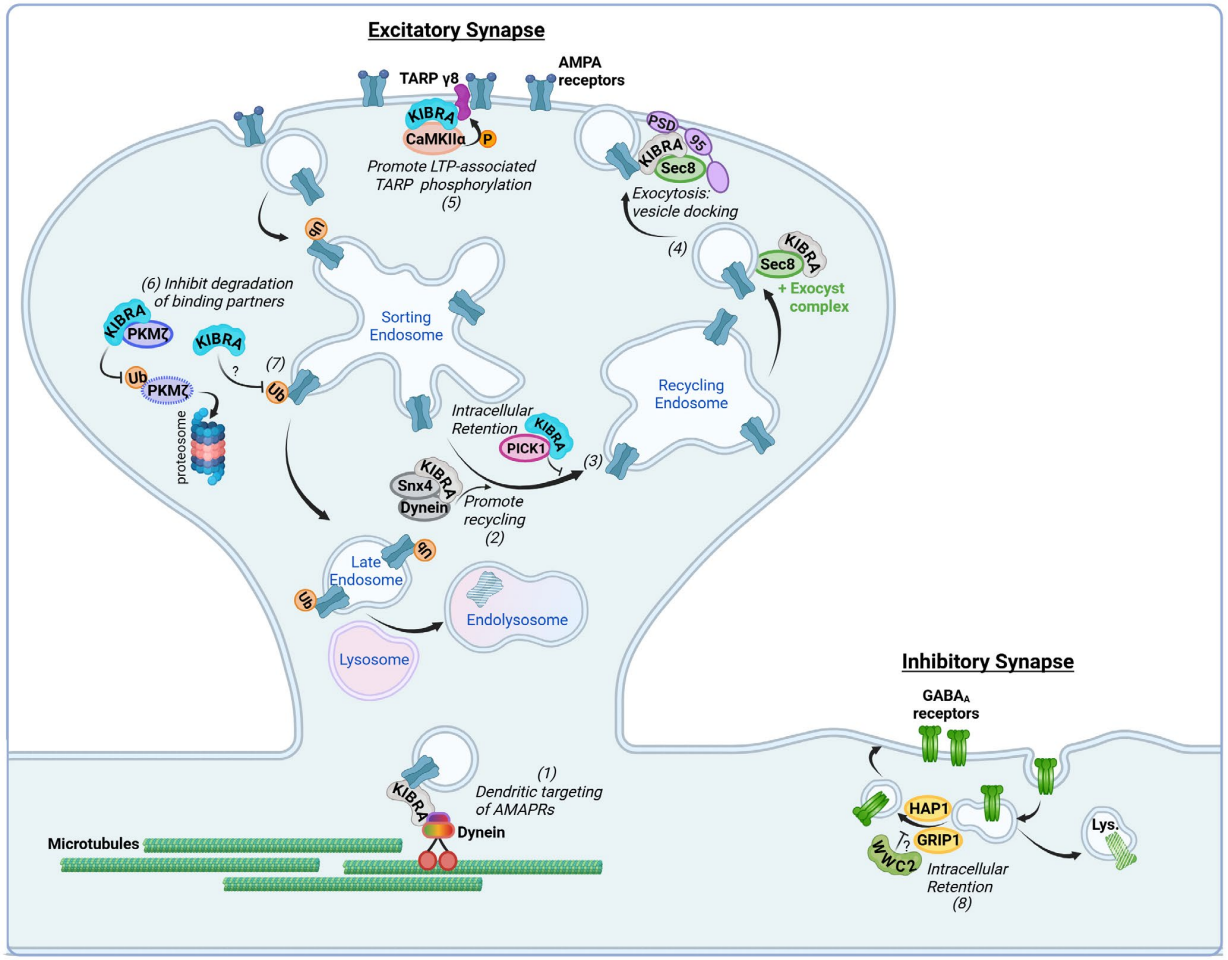
Model summarizing potential mechanisms by which WWC proteins regulate trafficking of ionotropic neurotransmitter receptors. Complexes shown in gray (e.g., KIBRA, Snx4, Dynein) have been shown to regulate the indicated trafficking step for receptors other than AMPARs. Complexes in which KIBRA is gray and the KIBRA binding partner(s) are shown in color indicate a demonstrated role for the binding partner in AMPAR trafficking whereas the role for KIBRA in the indicated process is not yet known or has not been specifically shown for AMPARs. Complexes with KIBRA shown in teal reflect processes with evidence supporting a role for KIBRA in the indicated regulatory step. KIBRA and its binding partners are implicated in multiple steps of AMPAR trafficking and synaptic regulation. (1) The microtubule motor complex dynein facilitates dendritic targeting of AMPARs. KIBRA binds a component of the dynein motor (DYNLL1), though whether microtubule‐based transport of AMPARs is regulated by KIBRA is an open question. (2) Dynein and KIBRA, along with Snx4, promote trafficking of transferrin receptors to the endosomal recycling complex. Whether this complex also functions in neurons to promote AMPAR trafficking towards recycling endosomes is not yet known. (3) KIBRA and its binding partner PICK1 are implicated in retaining endocytosed GluA2, whereas KIBRA has been shown to promote insertion of GluA1‐containing AMPARs (4), suggesting that KIBRA function is influenced by interaction with distinct AMPAR regulatory complexes and highlighting the importance of understanding how KIBRA protein–protein interactions are regulated. (4) The mechanisms engaged by KIBRA to promote AMPAR (GluA1) surface expression have not been conclusively elucidated, but one potential mechanism is through regulation of the exocyst complex, which functions to traffic AMPARs to synaptic membranes and promote exocytosis. (5) KIBRA regulates TARPγ8 phosphorylation by CaMKIIα at a site implicated in promoting LTP, suggesting a potential mechanism for regulating activity‐induced AMPAR trafficking. (6) LTP also increases KIBRA interaction with the brain‐specific kinase PKMζ. How enhanced KIBRA‐PKMζ. interaction facilitates LTP maintenance in the mammalian brain is not clear, though KIBRA has been shown to protect PKMζ from degradation in the mammalian brain, and in marine mollusks KIBRA‐dependent stabilization of aPKCs promotes associative long‐term facilitation. Notably, KIBRA decreases ubiquitination and degradation of multiple binding partners, suggesting that recruiting deubiquitinating enzymes or sequestering binding partners from ubiquitin ligases may be a shared mechanism for regulating KIBRA binding partners. (7) Indirect evidence suggests that KIBRA may protect AMPARs from degradation, providing another mechanism by which KIBRA may regulate AMPAR trafficking and synaptic function. (8) In contrast to KIBRA, WWC2 regulates GABA_A_R expression at inhibitory synapses. Current evidence suggests that WWC2 acts as a negative regulator of GABA_A_R surface expression, and implicates WWC2 in HAP1/GRIP1‐dependent GABA_A_R recycling. Created in BioRender. https://BioRender.com/o0vsfof.

A number of KIBRA's binding partners regulate distinct aspects of trafficking through recycling endosomes (Figure 3), including PICK1 (retention of AMPARs in recycling endosomes) (Terashima et al. 2008; Madsen et al. 2012), SNX4 (targeting receptors to the endosomal recycling complex, away from lysosomes) (Traer et al. 2007), DYNLL1 (microtubule motor, trafficking of recycling endosomes to late endosomes) (Traer et al. 2007; Rayala et al. 2006; Aniento et al. 1993), and CAMDI (Rab11 activation) (Fukuda et al. 2019). Existing data supports a role for KIBRA in regulating endosomal recycling of AMPRs (Makuch et al. 2011; Heitz et al. 2016; Fukuda et al. 2019; Traer et al. 2007), but the precise mechanisms and AMPAR subunit specificity of such regulation by KIBRA remain open questions. KIBRA also interacts with members of the exocyst complex (Sec3 (Rosse et al. 2009), Sec8 (Makuch et al. 2011)) implicated in trafficking of AMPARs towards synapses (Gerges et al. 2006), providing an additional mechanism by which KIBRA may regulate AMPAR trafficking. Finally, KIBRA binds phospholipids in a Ca^2+^‐independent manner through its C2 domain (Duning et al. 2013; Posner et al. 2018) which may facilitate membrane association of KIBRA complexes (Lemmon 2008), though if and how the KIBRA C2 domain contributes to AMPAR trafficking is unknown.

Hints to an additional mechanism by which KIBRA may regulate activity‐induced AMPAR trafficking come from studies examining the function of KIBRA WW domain interactions. KIBRA is a key upstream regulator of the Hippo signaling pathway, which controls cell proliferation and survival, tissue homeostasis, and organ development (Zheng and Pan 2019). KIBRA WW domains interact with several proteins in the Hippo pathway containing proline‐rich sequences (e.g., PPxY), a common interaction motif throughout the Hippo pathway (Stepan et al. 2022; Xiao, Chen, Ji, and Dong 2011; Cao et al. 2023). While little is known about the function of Hippo signaling in synaptic or neuronal function, intriguing results from recent studies suggest that Hippo signaling complexes may compete with AMPAR regulatory complexes for interaction with KIBRA (Stepan et al. 2022; Stepan et al. 2024). Mutation of KIBRA WW domains decreases interaction with multiple Hippo signaling molecules (Stepan et al. 2022; Cao et al. 2023) and increases KIBRA interaction with AMPAR regulatory protein networks, an effect that is replicated by pharmacological treatment that decouples KIBRA from the Hippo‐pathway kinase LATS (Stepan et al. 2024). Overexpression of KIBRA WW domain mutants in the hippocampus also improves memory performance, further supporting a role for KIBRA‐mediated regulation of AMPARs in learning and memory (Stepan et al. 2022). Among the KIBRA interactome showing enhanced interaction with KIBRA WW domain mutants are the AMPAR auxiliary protein TARPγ8 (transmembrane AMPAR regulatory protein gamma 8) and CaMKIIα (Stepan et al. 2022). Phosphorylation of TARPγ8 by CaMKIIα promotes hippocampal LTP and underlies CaMKIIα‐dependent increases in AMPAR‐mediated synaptic transmission (Park et al. 2016). TARPγ8 phosphorylation at the LTP‐ and CaMKIIα‐regulated serine‐277 site is increased in cultured HC neurons overexpressing KIBRA WW domain mutants (Stepan et al. 2022), suggesting that scaffolding of TARPγ8 and CaMKIIα may be another mechanism by which KIBRA facilitates LTP‐induced AMPAR trafficking.

### 5.5 KIBRA Promotes Protein Stability of Its Binding Partners

Total AMPAR protein levels are reduced in the hippocampus following KIBRA deletion in mice (Mendoza et al. 2022). While the mechanism of such regulation is not yet known for AMPARs, KIBRA has been shown to protect multiple binding partners from ubiquitination and degradation, including PKMζ, Rab27a, LATS1/2, and AMOT (Vogt‐Eisele et al. 2014; Xiao, Chen, Ji, and Dong 2011; Cao et al. 2023; Song et al. 2019), suggesting that this may be a common mechanism by which KIBRA regulates members of its interactome. Ubiquitination directs AMPAR trafficking to lysosomes for degradation and regulates synaptic plasticity (Widagdo et al. 2015; Guntupalli et al. 2023; Schwarz et al. 2010), thus it is possible that regulation of AMPAR ubiquitination may contribute to KIBRA's role in AMPAR trafficking.

KIBRA‐mediated stabilization of aPKCs has been implicated in synaptic plasticity (Hu et al. 2017; Tsokas et al. 2024). KIBRA interacts with the catalytic subunit of atypical PKC isoforms through a C‐terminal region similar to the aPKC pseudosubstrate sequence (Büther et al. 2004; Yoshihama et al. 2011). In marine mollusks (Aplysia) KIBRA stabilizes PKM Apl‐III, and disrupting KIBRA‐PKM Apl‐III interaction blocks late‐phase associative LTF (Hu et al. 2017). Mammalian KIBRA also promotes stability of the aPKC isoform PKMζ, as demonstrated in rodent brain tissue lacking KIBRA or following heterologous expression in cell culture (Kauwe et al. 2024; Mendoza et al. 2022; Vogt‐Eisele et al. 2014; Tsokas et al. 2024). LTP has been shown to increase KIBRA‐PKMζ interaction, and interfering with KIBRA‐PKMζ interaction using peptides to block the PKMζ‐binding site on KIBRA or the KIBRA‐binding site on PKMζ reverses LTP and associative memory (Tsokas et al. 2024). These results suggest that activity‐induced KIBRA‐PKMζ interaction promotes synaptic plasticity and memory maintenance. How neural activity increases KIBRA‐PKMζ interaction and the precise mechanisms by which KIBRA‐PKMζ complexes regulate synaptic function remain open questions. KIBRA inhibits aPKC activity in epithelial cells (Yoshihama et al. 2011), but whether KIBRA impacts PKMζ kinase activity in neurons is unknown. Further investigation is also required to determine whether and when KIBRA‐mediated stabilization of PKMζ protein is an essential regulator of synaptic plasticity in the mammalian brain. While KIBRA deletion in adult mice impairs synaptic plasticity and AMPAR expression, KIBRA deletion induced in juvenile mice does not impair synaptic plasticity or AMPAR expression (Mendoza et al. 2022). In contrast, PKMζ expression levels are similarly decreased in both adult‐induced and juvenile‐induced KIBRA cKO mice, indicating that KIBRA‐mediated PKMζ stabilization is not essential for synaptic plasticity in the juvenile brain (Mendoza et al. 2022). Further investigation is required to determine if the dissociation between KIBRA‐regulated PKMζ stabilization and KIBRA‐mediated AMPAR regulation and synaptic plasticity reflects developmental differences in synaptic plasticity mechanisms.

Numerous proteins within the KIBRA interactome facilitate AMPAR trafficking and synaptic plasticity (Figure 2B). A major goal for future research will be to determine how and when KIBRA engages different members of its interactome to promote synaptic plasticity, and whether distinct types of neuronal activity or cellular localization impact the composition and function of KIBRA‐organized protein complexes. How KIBRA‐dependent mechanisms of synaptic and cellular regulation change across development and aging is poorly understood. While only a few studies have examined age‐dependent changes in KIBRA function, KIBRA was shown to regulate maintenance of extrasynaptic AMPAR content and LTP‐induced AMPAR expression in the adult hippocampus but not in hippocampal tissue from juvenile mice, paralleling the adult‐selective role for KIBRA in synaptic plasticity (Makuch et al. 2011; Mendoza et al. 2022; Blanque et al. 2015). The mechanisms underlying age‐dependent differences in KIBRA function at synapses, and whether this phenomenon is specific to the hippocampus are questions that warrant further investigation.

## 6 Regulation of KIBRA Function

### 6.1 Regulation by Neuronal Activity

Despite accumulating evidence demonstrating a key role for KIBRA in regulating synaptic, circuit, and behavioral adaptations in response to neuronal activity and experience (Tracy et al. 2016; Makuch et al. 2011; Mendoza et al. 2022; Quigley et al. 2023; Heitz et al. 2016; Vogt‐Eisele et al. 2014; Ji et al. 2019; Tsokas et al. 2024), little is known about how KIBRA function, expression, and/or localization are themselves regulated by activity. Recent work suggests that KIBRA protein expression is increased in hippocampal dendrites following LTP induction (Tsokas et al. 2024), and elevated KIBRA protein expression was reported in the prefrontal cortex following learning (Wang, Liu, et al. 2014). Consistent with the idea that release of KIBRA from Hippo pathway interactions can promote association with synaptic AMPAR regulatory complexes, LTP decreases KIBRA interaction with the Hippo pathway kinase LATS1 (Stepan et al. 2022) and increases interaction with PKMζ (Tsokas et al. 2024). The mechanisms by which neuronal activity increases KIBRA protein expression or regulates KIBRA's protein interactions remain to be determined.

### 6.2 Posttranslational Modification of KIBRA

Multiple posttranslational modifications of KIBRA have been identified, including phosphorylation, palmitoylation, and ubiquitination (Table 2), many of which are associated with regulation of cell cycle progression. Whether these or other as yet unidentified posttranslational modifications occur in neurons, are regulated by neuronal activity, or affect synaptic or brain function are open questions. Among the kinases shown to phosphorylate KIBRA, ERK, RSK, and aPKCs are implicated in several forms of synaptic plasticity (Tsokas et al. 2024; Thiels et al. 2002; Di Cristo et al. 2001; Kanterewicz et al. 2000; Sanderson et al. 2016; Liu et al. 2020; Ren et al. 2013). Thus, determining if KIBRA phosphorylation by these kinases is regulated by neuronal activity and/or affects KIBRA function in neurons should be among the priorities for future studies.

### 6.3 Targeting to Dendrites/Synapses

KIBRA protein shows enriched localization to excitatory synapses (Makuch et al. 2011; Johannsen et al. 2008) but conflicting evidence exists regarding the molecular determinants required for targeting KIBRA to synapses. Work from Ji et al. suggests that interaction with DDN targets KIBRA to excitatory synapses (Ji et al. 2019). The N‐terminal WW domains of KIBRA interact with tandem PPxY motifs in DDN (Ji et al. 2019), which is itself recruited to excitatory synapses through association with the postsynaptic scaffold S‐SCAM/MAGI (Kremerskothen et al. 2006). Incubating neurons with a peptide encompassing the KIBRA‐interacting PPxY tandem sequence from DDN substantially decreased KIBRA localization to dendritic shafts and spines (Ji et al. 2019), suggesting that interaction with DDN (or other WW domain‐mediated interactions that are disrupted by the DDN peptide) recruits KIBRA to excitatory synapses. However, Kauwe et al. found that an N‐terminal KIBRA fragment encompassing just the WW domains (amino acids 1–86) showed substantially reduced expression in dendritic spines compared to full‐length KIBRA (Kauwe et al. 2024), indicating that WW domain‐mediated interactions alone are not sufficient to maintain synaptic localization of KIBRA. In contrast, dendritic spine localization of a C‐terminal KIBRA fragment containing the aPKC‐binding site, putative coiled‐coil domain, and PDZ ligand was comparable to full‐length KIBRA (Kauwe et al. 2024). Data from Stepan et al. similarly suggest that WW domain interactions may not be critical for synaptic localization of KIBRA (Stepan et al. 2022). KIBRA WW domain mutation (P37A/P84A) impairs interaction with PPxY motifs in numerous proteins (Kremerskothen et al. 2003; Duning et al. 2008; Stepan et al. 2022; Cao et al. 2023), though the effect of this specific mutation on KIBRA‐DDN interaction has not been examined. These KIBRA WW domain mutants show increased interaction with synaptic proteins, including the core excitatory synapse scaffold PSD‐95 (Stepan et al. 2022), arguing against a requirement for WW domain‐mediated interactions in promoting synaptic localization of KIBRA. As KIBRA forms a complex with numerous synaptically‐localized proteins (Figure 2B), further detailed structure–function studies will be needed to determine the mechanisms responsible for KIBRA localization to excitatory synapses.

### 6.4 Regulation of Biomolecular Condensates

Biomolecular condensates are membraneless compartments commonly formed through liquid–liquid phase separation (LLPS) that can concentrate and segregate biomolecules (Banani et al. 2017). Emerging evidence suggests that biomolecular condensates may serve a key organizing function in both pre and postsynaptic terminals (Choi et al. 2024; Hosokawa and Liu 2021). LLPS promoted by synaptic scaffolding proteins is implicated as a mechanism supporting formation of the highly‐condensed excitatory postsynaptic densitity (Zeng et al. 2016; Zeng et al. 2018) as well as a mechanism for segregating proteins in postsynaptic nanodomains (Hosokawa et al. 2021) and clustering synaptic AMPA receptors (Zeng et al. 2019). KIBRA has been shown to induce biomolecular condensates in vitro and in non‐neuronal cells to regulate Hippo signaling (Ji et al. 2019; Wang et al. 2022; Bonello et al. 2023). KIBRA's ability to induce LLPS is dependent on both its central and C‐terminal coiled‐coil domains (Wang et al. 2022; Bonello et al. 2023). Interestingly, recent work showed that KIBRA can form large protein clusters dependent on its coiled‐coil domains that recruit/segregate key excitatory synaptic proteins (PICK1/AMPARs) (Shao and Volk 2025). Whether KIBRA contributes to postsynaptic organization and signaling compartmentalization through LLPS in neurons is an interesting question for future investigations.

Although KIBRA's role in memory and synaptic plasticity is well‐documented, surprisingly little is known about the regulatory mechanisms that modulate KIBRA activity, expression levels, and localization in neurons and other brain cells. Insights from other tissues offer candidate pathways to test in the brain, but brain and cell type‐specific mechanisms of KIBRA regulation represent a significant area for discovery.

## 7 WWC Proteins Regulate Ionotropic Neurotransmitter Receptors at Distinct Classes of Synapses

In mammals the WW and C2 domain‐containing protein family consist of 3 homologs, KIBRA (*a.k.a*. WWC1), WWC2, and WWC3, with the exception of mice which express only WWC1 and WWC2 (Wennmann et al. 2014). Human KIBRA shares 49% sequence identity with WWC2 and 40% sequence identity with WWC3 (Wennmann et al. 2014). Each homolog possess the eponymous WW and C2 domains in addition to coiled‐coil regions and a C‐terminal PDZ ligand (Zhang et al. 2014). All three WWC proteins regulate Hippo pathway signaling (Wennmann et al. 2014). Compared to KIBRA, much less is known about the functions of WWC2 in the brain, and the physiological role of WWC3 in the brain is unknown. WWC2 gene variants and altered gene expression have been associated with neurodevelopmental and neurodegenerative disorders (Iossifov et al. 2014; Fromer et al. 2016; Runne et al. 2007), but the impact of WWC2 dysfunction on cognition remains to be determined. Whereas KIBRA and WWC2 show largely non‐overlapping expression in non‐neuronal brain cells (KIBRA is expressed in astrocytes whereas WWC2 is expressed in oligodendrocytes, vascular leptomeningeal cells, and endothelial cells), KIBRA and WWC2 are expressed in overlapping populations of neurons (Dunham et al. 2024). It has long been assumed that KIBRA and WWC2 perform similar functions at synapses (Makuch et al. 2011; Stepan et al. 2024). However, recent work suggests that this is unlikely to be the case. Rather, it appears that whereas KIBRA regulates AMPAR trafficking and expression at excitatory synapses (Makuch et al. 2011; Mendoza et al. 2022; Heitz et al. 2016), WWC2 localizes to inhibitory synapses and regulates GABA_A_R trafficking (Dunham et al. 2024; Uezu et al. 2016) (Figure 3). Specifically, WWC2 functions as a negative regulator of GABA_A_R trafficking such that membrane/surface GABA_A_R expression and inhibitory synaptic transmission are increased in hippocampal tissue from mice lacking WWC2 (Dunham et al. 2024). Much remains to be determined regarding the mechanisms by which WWC2 regulates GABA_A_R trafficking, but initial results point to a role for regulation through HAP1 and GRIP1, which function to promote trafficking of endosomal GABA_A_Rs back to the plasma membrane (Dunham et al. 2024; Twelvetrees et al. 2019, 2010).

Several key areas require further investigation regarding the synaptic function of WWC2. These include determining if the synaptic function of WWC2 is differentially regulated across postnatal development as is the case for KIBRA (Makuch et al. 2011; Mendoza et al. 2022), identifying the mechanisms by which KIBRA and WWC2 are preferentially targeted to excitatory and inhibitory synapses, respectively, and determining whether WWC2 regulates activity‐dependent plasticity of inhibitory synaptic transmission in addition to its role in basal synaptic transmission and GABA_A_R trafficking.

## 8 KIBRA and WWC2 Regulate Neuronal Morphology

In contrast to their divergent roles in excitatory and inhibitory synapse function, KIBRA and WWC2 both promote dendritic arborization. Knockout of either KIBRA or WWC2 decreases dendritic complexity (Heitz et al. 2016; Dunham et al. 2024), suggesting that they perform complementary rather than redundant functions in promoting dendritic arborization. KIBRA and WWC2 can form heterodimers (Wennmann et al. 2014), so the lack of redundancy could result from a requirement for signaling unique to KIBRA‐WWC2 heterodimers, or it could indicate a minimum dosage requirement for WWC proteins in general. The mechanisms by which WWC proteins regulate neuronal morphology are currently unknown. However, multiple Hippo pathway components, including the KIBRA and WWC2‐interacting proteins Lats1/2 and AMOT, regulate dendritic arborization in a manner similar to KIBRA and WWC2 (Rojek et al. 2019; Emoto et al. 2006), raising the possibility that KIBRA and WWC2 may engage components of the Hippo signaling pathway to promote dendritic arborization.

WWC proteins have also been shown to regulate dendritic spine morphology. In juvenile KIBRA KO mice, pyramidal neurons in hippocampal area CA1 and layer 5 of the neocortex exhibit an increased proportion of filopodia‐like spines, with no change in spine density (Blanque et al. 2015). Deletion of both KIBRA and WWC2 resulted in decreased spine density in the prefrontal cortex and hippocampal dentate gyrus (Cao et al. 2023). Interestingly, KIBRA and WWC2 interact with the Hippo pathway regulator AMOT, and overexpression of AMOT partially rescues the decreased dendritic spine density observed in mice lacking both KIBRA and WWC2 (Cao et al. 2023). How loss of WWC2 alone affects dendritic spine structure or density remains to be determined. Additionally, whether KIBRA and/or WWC2 regulate activity‐induced changes in dendritic spines is an open question.

Though Hippo signaling is a promising candidate mechanism for regulation of neuronal morphology, whether Hippo signaling is engaged by KIBRA and/or WWC2 to promote dendritic arborization or regulate dendritic spines remains to be determined, and there are other pathways through which KIBRA could affect neuronal structure (Figure 2). With respect to developmental timing, alterations in dendritic spine structure are observed in juvenile (P21) KIBRA KO mice, before synaptic plasticity or AMPAR trafficking deficits emerge (Makuch et al. 2011; Mendoza et al. 2022; Blanque et al. 2015). The impact of adult‐induced KIBRA deletion on dendritic spine structure and dendritic arborization has not been evaluated; therefore, it is not clear if KIBRA‐mediated signaling contributes to developmental establishment versus maintenance of dendritic morphology, nor is it known if altered dendritic or spine morphology contributes to the synaptic and network plasticity deficits observed upon adult‐induced KIBRA deletion.

## 9 KIBRA and Its Interactome are Disrupted in Cognitive Disorders

### 9.1 Neurodegenerative Disorders

Reduced KIBRA protein expression in the brain is associated with worse CDR and higher levels of pathological tau species (acetylated or phosphorylated tau) in patients diagnosed with tauopathies (Alzheimer's disease, Pick's disease) (Kauwe et al. 2024; Tracy et al. 2016). Studies in animal models of tauopathy (Kauwe et al. 2024; Tracy et al. 2016) and Alzheimer's disease (Stepan et al. 2024) and work in aged mice (Stepan et al. 2024) suggest that manipulating KIBRA expression or function may be sufficient to improve memory performance in age‐ and neurodegenerative disorder‐related cognitive decline. Overexpressing the C‐terminal region of KIBRA encompassing the aPKC binding site and likely C‐terminal coiled‐coil structure rescues memory deficits in mice expressing mutant tau protein (Kauwe et al. 2024), whereas pharmacological treatment with a MST1/2 inhibitor that increases KIBRA interaction with AMPAR regulatory complexes facilitates memory in aged mice and in mice expressing familial Alzheimer's disease mutations in the amyloid β protein (Stepan et al. 2024). While these studies are promising, further investigation is needed to support the utility of such approaches across different forms of age‐related memory impairment and to determine the mechanisms and duration of KIBRA‐based memory improvement.

### 9.2 Neurodevelopmental Disorders

Dysregulation of glutamatergic synapses and neuronal morphology are shared features across multiple neurodevelopmental disorders (NDDs) (Volk et al. 2015; Goikolea‐Vives and Stolp 2021). As a scaffold, organization of protein complexes is a core mechanism of KIBRA protein function. Notably, a substantial proportion of the KIBRA interactome is associated with NDDs (Kos et al. 2016; Fromer et al. 2016; Nomura et al. 2016; Lauriat et al. 2006; Hakak et al. 2001; Hou et al. 2016; Voineagu et al. 2011) in addition to regulation of excitatory synapses and neuronal morphology (Figure 2), suggesting that signaling regulated by KIBRA complexes may represent convergent vulnerability to NDDs.

KIBRA polymorphisms and gene expression have been associated with a number of neuropsychiatric diseases and NDDs (Figure 2C, Table 1). Exome sequencing identified *KIBRA* as a high‐confidence risk gene for Tourette Syndrome (TS) (Willsey et al. 2017; Wang et al. 2018), a developmental neuropsychiatric syndrome characterized by motor and vocal tics (Schilke et al. 2022; Leisman and Sheldon 2022). Subsequent work using a mouse knock‐in model harboring the Tourette‐associated W88C KIBRA mutation revealed a role for KIBRA in striatal neurodevelopment and elevated dopamine signaling in the striatum (Lv et al. 2025), hallmarks of neuropathology in TS (Schilke et al. 2022; Leisman and Sheldon 2022). KIBRA^W88C^ knockin (KI) mice show increased cortico‐striatal synaptic transmission, and medium spiny neurons (MSNs) in the striatum exhibit an increased frequency of spontaneous EPSCs and a decreased frequency of spontaneous IPSCs. These data suggest that KIBRA^W88C^ mutation results in increased cortico‐striatal input and an enhanced E/I synaptic ratio in striatal MSNs. The mechanisms by which KIBRA^W88C^ regulates synaptic transmission in the striatum remain to be determined, though current evidence suggests it is not through regulating postsynaptic AMPARs (as indicated by unaltered miniature and spontaneous EPSC amplitude). Interestingly, despite decreased protein expression due to enhanced proteosomal degradation of KIBRA^W88C^, Hippo signaling is upregulated in KIBRA^W88C^ KI mice as indicated by elevated LATS and YAP phosphorylation (Lv et al. 2025), suggesting that this mutation causes a gain‐of‐function, at least in terms of regulating Hippo signaling. Consistent with demonstrated roles for KIBRA and other Hippo signaling components in promoting dendritic arborization (Heitz et al. 2016; Rojek et al. 2019; Emoto et al. 2006), striatal MSNs from KIBRA^W88C^ KI mice exhibit enhanced dendritic complexity (Lv et al. 2025). Reintroduction of wild type (WT) KIBRA in P0 KIBRA^W88C^ KI mice rescues hippo signaling, dendritic complexity, and elevated neuronal activity in the striatum and primary motor cortex (measured by c‐Fos + neurons), in addition to rescuing elevated repetitive motor behaviors (Lv et al. 2025). In contrast, reintroduction of WT KIBRA in two‐month‐old KIBRA^W88C^ mice fails to rescue TS‐relevant behaviors, supporting an important role for KIBRA in early postnatal neurodevelopment (Lv et al. 2025) that is temporally distinct from its role in regulating synaptic plasticity in the mature brain (Makuch et al. 2011; Mendoza et al. 2022). KIBRA^W88C^ KI mice show normal basal excitatory synaptic transmission and AMPAR protein content at hippocampal synapses, which is similar to the unaffected basal synaptic transmission observed in constitutive or conditional KIBRA KO mice (Makuch et al. 2011; Mendoza et al. 2022; Lv et al. 2025). However, this contrasts with data showing that the W88C mutation disrupts KIBRA interaction with DDN (at least in vitro), and that acute peptide‐based disruption of KIBRA‐DDN interaction impairs basal excitatory synaptic transmission and targeting of AMPR to synapses (Ji et al. 2019). Thus, it is possible that the W88C mutation does not affect KIBRA‐DDN interaction in vivo, or that neurons are able to compensate for chronic loss of KIBRA‐DDN interaction. While it is not known if KIBRA^W88C^ affects hippocampal synaptic plasticity, the finding that KIBRA^W88C^ KI mice show normal learning and memory (Lv et al. 2025) suggests that KIBRA^W88C^ does not disrupt adaptive hippocampal function.

**TABLE 1:** The KIBRA interactome, references and interaction details related to Figures 1 and 2.

Protein	(A) related to Figures 1 and 2A		^(B)^	^(C)^
	Mechanism of interaction with KIBRA	Evidence supporting interaction	Figure 2B	Figure 2C
AKAP5	Mechanism unknown (increased association with KIBRA WW domain mutant)	Co‐IP: OE KIBRA FL and WW domain point mutants in neurons with endogenous AKAP5 (Stepan et al. 2022)	(Diering et al. 2014)	
AMOT	Direct: KIBRA WW domain, AMOT PPXY sequence	Co‐IP: domain mutants in heterologous cells, endogenous proteins from brain tissue (Cao et al. 2023)	(Rojek et al. 2019)	(Parikshak et al. 2016; Gandal et al. 2018)
aPKCs (ι/λ, ζ)	Direct: KIBRA C‐terminal sequence, aPKC catalytic domain	Y2H: domain mutants (Büther et al. 2004), co‐IP: domain mutants in heterologous cells (Vogt‐Eisele et al. 2014), fluorescence complementation: FL KIBRA and PKMζ in neurons (Tsokas et al. 2024), co‐IP: endogenous protein from brain tissue (Yoshihama et al. 2009)	(Hu et al. 2017; Tsokas et al. 2024; Ren et al. 2013)	(Gandal et al. 2018)
ATM kinase	Direct: ATM kinase phosphorylates KIBRA C‐terminus	in vitro and in vivo kinase assay, co‐IP: FL proteins in heterologous cells (Mavuluri et al. 2016)		
AURKA	Direct: KIBRA central CC region regulated by S539 phosphorylation, AURKA C‐terminal portion of kinase domain is required but kinase domain alone is not sufficient	in vitro and in vivo kinase assay, co‐IP: domain mutants in heterologous cells (Xiao, Chen, Ji, Volle, et al. 2011)		
β‐TrCP1/2	Direct: KIBRA 601–700, interaction inhibited by KIBRA^S631A^ mutation. KIBRA‐β‐TrCP1/2 interaction is enhanced by NF2	Proximity‐dependent biotin identification: FL proteins, co‐IP: FL and domain mutants in heterologous cells (Wang, Zhu, et al. 2025)		(De Rubeis et al. 2014; Ruzzo et al. 2019)
CAMDI	Direct: KIBRA C‐terminal CC domain, CAMDI CC domains 2 and 3	Y2H: CAMDI CC region retrieved KIBRA C‐terminal CC domain, co‐IP: domain mutants in heterologous cells (Fukuda et al. 2019)	(Fukuda et al. 2019)	
CaMKIIα	Mechanism unknown (increased association with KIBRA WW domain mutant)	Co‐IP: OE KIBRA FL and WW domain point mutants in neurons with endogenous CaMKIIα (Stepan et al. 2022)	(Nicoll and Schulman 2023)	(Iossifov et al. 2014)
CASK	Mechanism unknown (increased association with KIBRA WW domain mutant)	Co‐IP: OE KIBRA FL and WW domain point mutants in neurons with endogenous CASK (Stepan et al. 2022)	(Gao et al. 2019)	(Iossifov et al. 2014)
CDC14A/B	Direct: KIBRA CDK1 phosporylation site mutants decrease interaction with CDC14B, CDC14A/B catalytically inactive mutant increases interaction with KIBRA	in vitro and in vivo phosphatase assays, co‐IP: point mutants in heterologous cells (Ji et al. 2012)		(Fromer et al. 2016)
CIT	Direct: KIBRA WW domains, CIT C‐terminal PPTY sequence	Co‐IP: domain mutants in heterologous cells (Wang, Li, et al. 2014)	(Camera et al. 2008; Repetto et al. 2014)	
CRB3	Mechanism unknown	Co‐IP: FL OE and endogenous protein in epithelial cell lines (Mao et al. 2018)		
DDN	Direct: KIBRA: WW domains, DDN PPXY motifs	Y2H: FL proteins (Kremerskothen et al. 2003), in vitro binding using ITC assay with purified domain and point mutants (Ji et al. 2019)	(Ji et al. 2019)	(Hou et al. 2016)
DDR1	Direct: KIBRA WW domains, DDR1 PPPY	Co‐IP: OE FL and domain mutants in heterologous cells (Hilton et al. 2008)		(Gandal et al. 2018)
DNAI1	Mechanism unknown (increased association with KIBRA WW domain mutant)	Co‐IP: OE KIBRA FL and WW domain point mutants in neurons with endogenous DNAI1 (Stepan et al. 2022)		
DNM1	Mechanism unknown (increased association with KIBRA WW domain mutant)	Co‐IP: OE KIBRA FL and WW domain point mutants in neurons with endogenous DNM1 (Stepan et al. 2022)	(Fà et al. 2014)	(Parikshak et al. 2016; Gandal et al. 2018)
DYNLL1,2	Direct, domains mediating interaction not known	in vitro pull down: FL purified protein, co‐IP: FL protein in heterologous cells (Rayala et al. 2006)	(Kapitein et al. 2010)	
FEZ1	Direct: KIBRA C‐terminal fragment containing CC domain, FEZ1 CC domain	Y2H: FEZ1 and KIBRA C‐terminus with CC domain, in vitro pull down: purified protein fragments (Assmann et al. 2006)	(Chua et al. 2021)	(Fromer et al. 2016)
GluA1	Mechanism unknown	Co‐IP: FL KIBRA, FL and C‐terminus of GluA1 in heterologous cells and neurons, endogenous proteins from brain tissue (Shao and Volk 2025; Makuch et al. 2011; Heitz et al. 2016)	(Whitlock et al. 2006; Jensen et al. 2003; Shepherd and Huganir 2007; Lee et al. 2003)	(*Nature* 2014; Geisheker et al. 2017)
GluA2	Indirect: PICK1 facilitates KIBRA‐GluA2 interaction	Co‐IP: FL proteins in heterologous cells, endogenous protein from brain tissue (Shao and Volk 2025; Makuch et al. 2011)	(Whitlock et al. 2006; Cao et al. 2018; Shepherd and Huganir 2007)	(Parikshak et al. 2016; Gandal et al. 2018; De Rubeis et al. 2014; RK et al. 2017; Salpietro et al. 2019)
GRIP1	Mechanism unknown (increased association with KIBRA WW domain mutant)	Co‐IP: OE KIBRA FL and WW domain point mutants in neurons with endogenous GRIP1, endogenous proteins from brain tissue (Makuch et al. 2011; Stepan et al. 2022)	(Dong et al. 1997; Mao et al. 2010)	(Mejias et al. 2011)
histone H3	Direct: KIBRA glutamate‐rich region	Far Western with purified protein, co‐IP: KIBRA domain mutant in heterologous cells (Rayala et al. 2006)		
KIAA0513	Mechanism unknown, likely direct	Y2H: KIAA0513 N‐terminus, co‐IP: FL proteins in heterologous cells (Lauriat et al. 2006)		
KIBRA	C2‐like domain and N‐terminal CC domains can support KIBRA dimerization, additional interactions may also support multimerization	Y2H: FL, domain deletions (Johannsen et al. 2008; Tona et al. 2024), co‐IP: FL and domain deletions in heterologous cells (Bates et al. 2009; Erdmann et al. 2007)	(Makuch et al. 2011; Mendoza et al. 2022; Heitz et al. 2016)	(Kos et al. 2016; Parikshak et al. 2016)
KIF5B	Mechanism unknown (increased association with KIBRA WW domain mutant)	Co‐IP: OE KIBRA FL and WW domain point mutants in neurons with endogenous KIF5B (Stepan et al. 2022)	(Zhao et al. 2020)	
LATS1/2	Direct: KIBRA WW domains, LATS1/2 PPPY sequence and C‐terminal kinase domain	Co‐IP: OE FL and domain mutants in heterologous cells and neurons (Stepan et al. 2022; Xiao, Chen, Ji, and Dong 2011)		
MPP2	Mechanism unknown (increased association with KIBRA WW domain mutant)	Co‐IP: OE KIBRA FL and WW domain point mutants in neurons with endogenous MPP2 (Stepan et al. 2022)	(Schwenk et al. 2012)	(Gandal et al. 2018)
MYO6	Mechanism unknown (increased association with KIBRA WW domain mutant)	Co‐IP: OE KIBRA FL and WW domain point mutants in neurons with endogenous MYO6 (Stepan et al. 2022)	(Wagner et al. 2019)	
NCAM1	mechanism unknown (increased association with KIBRA WW domain mutant)	co‐IP: OE KIBRA FL and WW domain point mutants in neurons with endogenous NCAM1 (Stepan et al. 2022)	(Muller et al. 1996)	(Mullins et al. 2021)
NF2	Direct: KIBRA: N and C‐terminus are sufficient for interaction whereas central region containing C2‐domain is not	Co‐IP: OE of domain mutants and endogenous protein in cultured cells, split‐TEV assay (Yu et al. 2010; Genevet et al. 2010)	(Schulz et al. 2010)	
NRXN1	Mechanism unknown (increased association with KIBRA WW domain mutant)	Co‐IP: OE KIBRA FL and WW domain point mutants in neurons with endogenous NRXN1 (Stepan et al. 2022)	(Singh et al. 2016; Pak et al. 2015)	(Mullins et al. 2021)
PAR3	Mechanism unknown	Co‐IP: OE FL proteins in heterologous cells, endogenous protein in kidney cell line (Yoshihama et al. 2011)	(Voglewede et al. 2024)	(Gandal et al. 2018)
PAR6	Mechanism unknown	Co‐IP: OE FL proteins in heterologous cells, endogenous protein in kidney cell line (Yoshihama et al. 2011)		(Gandal et al. 2018)
PATJ	Direct: KIBRA C‐terminal PDZ ligand (ADDV), PATJ PDZ domain 8	Y2H: domain mutants, co‐IP: OE FL and domain mutants in heterologous cells, GST pulldown: recombinant protein (Duning et al. 2008)		(Yousaf et al. 2020; Kenny et al. 2014)
PACSIN1	Mechanism unknown (increased association with KIBRA WW domain mutant)	Co‐IP: OE KIBRA FL and WW domain point mutants in neurons with endogenous PACSIN (Stepan et al. 2022)	(Anggono et al. 2013; Widagdo et al. 2016)	
PICK1	Likely direct: KIBRA central CC domain is sufficient in Y2H assay but multiple domains can support interaction, PICK1 BAR domain	Y2H, co‐IP: FL and domain mutants in heterologous cells, endogenous proteins from brain tissue (Shao and Volk 2025; Makuch et al. 2011)	(Volk et al. 2010)	(Parikshak et al. 2016; Gandal et al. 2018)
PP1	Direct: KIBRA S539A increases interaction, PP1 dephosphorylates KIBRA, DN PP1 does not interact with KIBRA	in vivo and in vitro phosphatase assays, co‐IP: FL and mutant proteins in heterologous cells (Xiao, Chen, Ji, Volle, et al. 2011)	(Foley et al. 2021)	(Gandal et al. 2018)
PSD‐95	Mechanism unknown (increased association with KIBRA WW domain mutant)	Co‐IP: OE KIBRA FL and WW domain point mutants in neurons with endogenous PSD‐95 (Stepan et al. 2022)	(Won et al. 2017)	(Wang et al. 2020; Stessman et al. 2017)
PTPN14	Direct: KIBRA WW domains, PTPN14 tandem PPPY/PPEY motifs	Co‐IP: FL and domain mutants in heterologous cells, ITC with isolated domains (Wang, Li, et al. 2014; Lin et al. 2019; Wilson et al. 2014)		
PTPN21	Direct: KIBRA WW domains, PTPN21 tandem PPSY/TPDY, TQVY/PPPY motifs	Co‐IP: FL proteins in heterologous cells, ITC with isolated domains (Wang, Li, et al. 2014; Lin et al. 2019)		(Gandal et al. 2018)
RAB27a	Mechanism unknown	Co‐IP: OE FL proteins in heterologous cells (Song et al. 2019)		
RSK1/2	Direct: KIBRA T929A/S947A blocks interaction with RSK1	in vitro kinase assay, in vivo kinase assay in heterologous cells, co‐IP: KIBRA WT and phospho mutants in heterologous cells (Yang et al. 2014)	(Liu et al. 2020)	(Gandal et al. 2018)
SAP97	Mechanism unknown (increased association with KIBRA WW domain mutant)	Co‐IP: OE KIBRA FL and WW domain point mutants in neurons with endogenous SAP97 (Stepan et al. 2022)	(Zheng et al. 2011)	(Krupp et al. 2017)
SEC3	Likely direct: KIBRA CC domains	Y2H: FL SEC3, four KIBRA fragments encompassing aa129‐526 (Rosse et al. 2009)		
SEC8	mechanism unknown	Co‐IP: endogenous proteins from brain tissue, OE FL protein in heterologous cells (Makuch et al. 2011; Rosse et al. 2009)	(Gerges et al. 2006)	
SHISA6	Mechanism unknown (increased association with KIBRA WW domain mutant)	Co‐IP: OE KIBRA FL and WW domain point mutants in neurons with endogenous SHISA6 (Stepan et al. 2022)	(Klaassen et al. 2016)	(Gandal et al. 2018)
SNX4	Likely direct, mechanism unknown	Y2H: FL SNX4, C‐term KIBRA, co‐IP: FL proteins in heterologous cells (Traer et al. 2007)	(Poppinga et al. 2024)	
SYNPO	Direct: KIBRA WW domains, SYNPO PPTY motif	Y2H: domain mutants, GST pulldown: FL and point mutants (Duning et al. 2008)	(Wu et al. 2024; Inglebert et al. 2024)	(Kos et al. 2016)
TARPγ8	Mechanism unknown (increased association with KIBRA WW domain mutant)	Co‐IP: OE KIBRA FL and WW domain point mutants in neurons with endogenous TARPγ8 (Stepan et al. 2022)	(Park et al. 2016; Sheng et al. 2018)	
WWC2	Mechanism unknown	Co‐IP: OE FL protein in heterologous cells (Wennmann et al. 2014)	(Dunham et al. 2024)	(Iossifov et al. 2014; Fromer et al. 2016)
TBC1D24	Direct: KIBRA C2 domain, TBC1D24 TLDc domain	Y2H: FL and domain deletions of KIBRA and TBC1D24, epilepsy‐associated point mutants of TBC1D24 (Tona et al. 2024)	(Lin et al. 2020; Falace et al. 2014)	(Banuelos et al. 2017; Balestrini et al. 2016)
14–3‐3‐β, θ, ζ	Mechanism unknown	Co‐IP: OE KIBRA FL and WW domain point mutants in neurons with endogenous 14–3‐3‐β (Stepan et al. 2022)	(Qiao et al. 2014)	(Iossifov et al. 2014; Gandal et al. 2018)
ZDHHC15	Direct: KIBRA C2 domain, ZDHHC15 catalytic domain	GST pulldown: purified proteins, co‐IP: domain mutants in heterologous cells, endogenous protein in ovarian cancer cell lines (Wang, Shen, et al. 2025)	(Shah et al. 2019)	(Mansouri et al. 2005)

*Note:* (A) Studies supporting interaction between KIBRA and the indicated protein, related to Figures 1 and 2A. (B) References indicating a role for the indicated protein in AMPAR trafficking, synaptic plasticity, synaptic transmission, and/or dendritic arborization, related to Figure 2B. (C) References indicating association with neurodevelopmental and neuropsychiatric disorders for the indicated protein, related to Figure 2C.

**TABLE 2:** Posttranslational modifications of KIBRA.

Phosphorylation	Kinase	Phosphatase	Supporting data	Function (synaptic/neuronal fxn.)	Ref.
Ser 539	Aurora‐A, ‐B	PP1	In vitro assay, in cultured cells	Cell cycle progression, NF2 interaction (unknown)	(Xiao, Chen, Ji, Volle, et al. 2011; Zhang et al. 2012)
Ser 542, Ser 548	ERK1/2		In vitro assay, in cultured cells	Cell proliferation (unknown)	(Yang et al. 2014)
Thr 929, Ser 947	RSK1/2		In vitro assay, in cultured cells	Cell proliferation, cell migration (unknown)	(Yang et al. 2014)
Ser 542, Ser 931	CDK1	CDC14A/B	In vitro assay, in cultured cells	Mitosis (unknown)	(Ji et al. 2012)
Ser 975, Ser 978	PKC/Mζ		In vitro assay, in cultured cells	Unknown (unknown)	(Büther et al. 2004)
Thr 1006	ATM kinase		In vitro assay, in cultured cells	DNA damage repair (unknown)	(Mavuluri et al. 2016)

Palmitoylation	Palmitoyl transferase	Supporting data	Function (in neurons)	Ref.
C705, C711	ZDHHC15	in cultured cells	promote KIBRA degradation, inhibit nuclear localization (unknown)	(Wang, Shen, et al. 2025)

Ubiquitination	E3 ubiquitin ligase	Supporting data	Function (in neurons)	Ref.
KIBRA^S631A^ decreases β‐TrCP1/2‐mediated ubiquitination	β‐TrCP1/2	in cultured cells	Promote KIBRA degradation (unknown)	(Wang, Zhu, et al. 2025)

In addition to a potential role in TS, exome sequencing of a quantitative trait locus for abstraction and mental flexibility identified a SNP (R250C) in exon 7 of *KIBRA* to significantly associate with schizophrenia (SCZ) risk (Kos et al. 2016). Finally, genome‐wide transcriptome analysis from a large cohort of individuals with Autism Spectrum Disorder (ASD) identified *KIBRA* as a hub gene in an ASD‐associated and developmentally‐regulated gene expression module (Parikshak et al. 2016).

## 10 Concluding Remarks and Outstanding Questions

Since its molecular identification and genetic association with human memory almost two decades ago, accumulated evidence supports a role for KIBRA across different scales of adaptive brain function, including memory, synaptic and network plasticity, and activity‐dependent regulation of AMPARs. The KIBRA interactome comprises an array of proteins that employ multiple mechanisms to regulate AMPARs and synaptic plasticity (Figure 3), highlighting the need for future studies to investigate whether molecularly distinct KIBRA complexes differentially modulate specific aspects of AMPAR trafficking and different types of synaptic plasticity. Furthermore, while KIBRA can interact with AMPAR subunits through multiple mechanisms (Shao and Volk 2025), it is unclear whether KIBRA regulates AMPARs in a subunit‐specific manner. Expanding the role of WWC proteins in synaptic regulation, WWC2 localizes to inhibitory synapses and regulates GABA_A_R trafficking. It remains unknown whether KIBRA and WWC2 utilize similar mechanisms to regulate AMPAR and GABA_A_R trafficking (relying on unique localization for their synapse‐specific effects), or if they engage different molecular pathways to manage neurotransmitter receptors.

KIBRA and WWC2 have also been implicated in the maturation of neuronal morphology and in neurodevelopmental pathology. KIBRA‐regulated Hippo pathway signaling is a candidate mechanism implicated in the development of neuronal morphology, whereas regulation of AMPAR trafficking by KIBRA is implicated in synaptic plasticity in mature neurons (Makuch et al. 2011; Mendoza et al. 2022; Heitz et al. 2016; Lv et al. 2025). Engagement with Hippo signaling molecules sequesters KIBRA from AMPAR complexes (Stepan et al. 2022; Stepan et al. 2024), suggesting that KIBRA's role in neurodevelopment may be mechanistically distinct from its role in synaptic regulation in mature neurons. However, significant crosstalk exists between pathways that regulate neuronal structure and synaptic function, and additional work is needed to determine the degree to which these processes are independently regulated. KIBRA was identified as a potential target for ameliorating aging and neurodegenerative disorder related cognitive decline, but considerable work remains to determine the therapeutic potential, underlying mechanisms, and potential side effects of such manipulations. For example, decoupling KIBRA from Hippo signaling molecules can improve cognition in the mature brain (Stepan et al. 2022, 2024), but evidence from TS‐associated KIBRA^W88C^ KI mice suggests that altering KIBRA‐dependent Hippo signaling may be detrimental to neurodevelopmental processes.

Little is known about how KIBRA function is regulated in neurons, or how memory‐associated *KIBRA* gene variants affect KIBRA expression or KIBRA function in humans. Therefore, investigating how KIBRA expression levels, synaptic targeting, and protein–protein interactions are regulated, particularly with respect to neuronal activity, represents important areas for future discovery. In addition, whether KIBRA‐induced biomolecular condensates are of functional significance in synaptic organization and neuronal physiology remains to be determined. Finally, gene expression data suggest that astrocytes and a subset of inhibitory neurons, most notably long‐range projecting somatostatin‐positive neurons, exhibit *Kibra* expression levels comparable to excitatory neurons in the hippocampus and cortex, but the function of KIBRA in these cell types is largely unknown. Data comparing striatal and hippocampal neurons in KIBRA^W88C^ KI mice suggest that KIBRA may serve distinct functions in different cell types or brain regions.

Lastly, the degree to which biological sex interacts with KIBRA function is poorly understood. While KIBRA has been shown to regulate synaptic plasticity in cohorts comprising male and female mice (Makuch et al. 2011; Mendoza et al. 2022), most studies on the role of KIBRA in synaptic plasticity have been conducted in male subjects (Heitz et al. 2016; Vogt‐Eisele et al. 2014; Ji et al. 2019; Tsokas et al. 2024). Similarly, the impact of KIBRA on learning, memory, and experience‐induced network dynamics has been explored primarily in male subjects (Makuch et al. 2011; Quigley et al. 2023; Heitz et al. 2016; Vogt‐Eisele et al. 2014; Stepan et al. 2022, 2024; Ji et al. 2019; Tsokas et al. 2024). Memory deficits were observed in a mixed population of male and female adult‐induced KIBRA KO mice (Mendoza et al. 2022), though this study did not include a comprehensive comparison between male and female cohorts. In TS‐associated KIBRA^W88C^ KI mice, the increased repetitive motor behavior and sensorimotor gating deficits observed in male mice were less pronounced in female mice, suggesting a potential interaction of sex with the effect of KIBRA mutations on motor behavior. In contrast, though similar proportions of male and female subjects are typically included in human genetic studies, sex × *KIBRA* genotype interactions have not been reported with respect to memory performance. Taken together, further investigation is warranted into potential interactions between sex and KIBRA's function in the brain.

In summary, KIBRA has emerged as a key regulator of memory and synaptic plasticity, with diverse roles in AMPAR trafficking and neuronal morphology. Future investigations should address gaps regarding mechanisms by which KIBRA is regulated, cell‐type‐specific functions of KIBRA, convergent and divergent mechanisms by which KIBRA and other WWC proteins regulate synaptic function and neuronal morphology across development and aging, and the impact of biological sex on these pathways.

## Author Contributions

**Lenora J. Volk:** conceptualization, funding acquisition, visualization, writing – original draft, writing – review and editing.

## Funding

National Institutes of Health Grant NIMH 1R01MH117149.

## Conflicts of Interest

The author declares no conflicts of interest.

## Data Availability

The author has nothing to report.
